# Topoisomeric Membrane-Active Peptides: A Review of the Last Two Decades

**DOI:** 10.3390/pharmaceutics15102451

**Published:** 2023-10-12

**Authors:** Adam Carrera-Aubesart, Maria Gallo, Sira Defaus, Toni Todorovski, David Andreu

**Affiliations:** 1 Department of Medicine and Life Sciences, Universitat Pompeu Fabra, 08003 Barcelona, Spain; adam.carrera@upf.edu (A.C.-A.); maria.gallo@upf.edu (M.G.); sira.defaus@upf.edu (S.D.); toni.todorovski@upf.edu (T.T.); 2 Department of Biotechnology, University of Rijeka, 51000 Rijeka, Croatia

**Keywords:** membrane-active peptides, cell-penetrating peptides, antimicrobial peptides, anticancer peptides, topoisomery, enantio, retro, retroenantio

## Abstract

In recent decades, bioactive peptides have been gaining recognition in various biomedical areas, such as intracellular drug delivery (cell-penetrating peptides, CPPs) or anti-infective action (antimicrobial peptides, AMPs), closely associated to their distinct mode of interaction with biological membranes. Exploiting the interaction of membrane-active peptides with diverse targets (healthy, tumoral, bacterial or parasitic cell membranes) is opening encouraging prospects for peptides in therapeutics. However, ordinary peptides formed by L-amino acids are easily decomposed by proteases in biological fluids. One way to sidestep this limitation is to use topoisomers, namely versions of the peptide made up of D-amino acids in either canonic (enantio) or inverted (retroenantio) sequence. Rearranging peptide sequences in this fashion provides a certain degree of native structure mimicry that, in appropriate contexts, may deliver desirable biological activity while avoiding protease degradation. In this review, we will focus on recent accounts of membrane-active topoisomeric peptides with therapeutic applications as CPP drug delivery vectors, or as antimicrobial and anticancer candidates. We will also discuss the most common modes of interaction of these peptides with their membrane targets.

## 1. Introduction

Membranes are biological structures that keep the internal contents (cytoplasm, nucleus, etc.) of a cell separate from the external medium, thus ensuring cell integrity. The distinctive constitutive feature of biological membranes is a phospholipid bilayer matrix that proteins, drugs, or any other molecule directed toward an intracellular destination must cross to reach their target [1,2,3]. Membrane-active agents perturb the basic features (e.g., permeability, fluidity, etc.) of the membrane and have a significant impact on cell function (e.g., transport, signaling, and/or integrity). A growing body of recent research has focused on various types of agents capable of modulating membrane physical properties and performance [4]. Among these, peptides stand out as particularly promising on account of their structural versatility that often translates into favorable physicochemical and biochemical properties. High target specificity, low toxicity, and (most often) low immunogenicity underpin the prospects of peptides in therapeutic areas [5,6] such as anti-infectives [7,8], cancer [9,10,11], cardiovascular [12,13], and Alzheimer’s disease [14,15], among others. 

Chirality is a key structural feature governing the action and specificity of bioactive peptides. In peptides, chirality stems from the presence of a stereogenic center at the α-carbon of every amino acid monomer (except Gly) of any peptide sequence (plus additional stereocenters at the β-carbons of Ile and Thr). While the vast majority of natural peptides are in an L-configuration at their α-carbon stereocenters, exceptions consisting of D-amino-acid-containing peptides in various families (arthropods, mollusks, amphibians, etc.) are known [16,17,18,19,20,21,22,23,24]. Peptides partially or totally made up of D-amino acids are predictably more stable in biological fluids than their all-L counterparts due to the resistance of peptide bonds with an adjoining α-carbon of a non-L configuration to protease hydrolysis [25,26,27]. This feature has been creatively exploited in recent times to boost the in vivo stability/performance of quite a few therapeutic peptides [28,29]. However, it is by no means a trivial tactic, as the switch from the L- to D-configuration at one or more stereocenters has an inevitable impact on the 3D structure of a peptide, which more often than not translates into partial or total loss of bioactivity. Tweaking peptide stereochemistry, therefore, inescapably requires significant effort in terms of, first, educated prediction and, second, the subsequent experimental evaluation of the biological outcomes of such structural manipulations [30]. 

**Figure 1:**
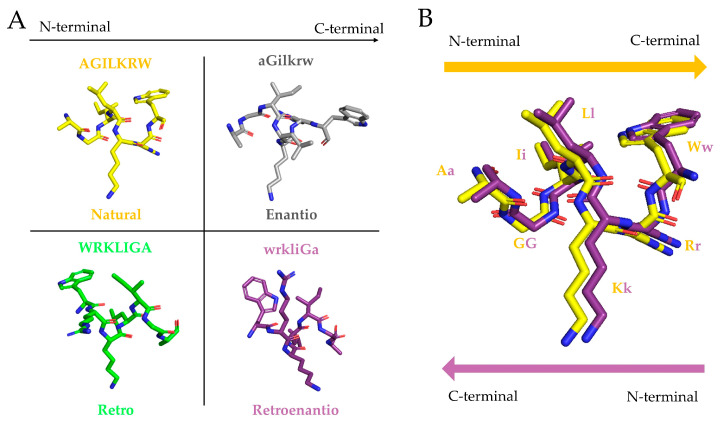
(**A**) Retro (WRKLIGA), enantio (aGilkrw), and retroenantio (wrkliGa) modifications of PepH3 (AGILKRW) [33] obtained using Pymol [34]. (**B**) Overlaying the canonical (yellow backbone, conventional left-to-right orientation, and N- to C-terminus layout) with the *re* (purple backbone, right-to-left orientation, N- to C-terminus layout) version shows that the side chains adopt similar orientations while the amide bonds are reversed.

Among the various approaches adopted by peptide medicinal chemists to overcome the in vivo vulnerability of bioactive peptides, this review focuses on tactics involving the conversion of bioactive peptide structures into their topoisomer versions. A generic definition of topoisomerism would correspond to composition-identical (hence isomeric) yet three-dimensionally distinct (hence topo-) variants of a molecule, with structural changes translating into different biological effects. In the peptide/protein field, this term has been preferentially applied to multiple disulfide peptides adopting different types of spatial folding as a result of alternative Cys pairings. Less frequently but also suitably, here and elsewhere [31], the term is used to describe all-D-amino acid counterparts of a peptide with either a conserved (the enantio version, abbreviated as *e* hereafter) or fully reversed (the retroenantio version, abbreviated as *re*) sequence relative to the canonical version. While all-D versions with, e.g., pairwise residue switches (or other maneuvers also preserving global composition) could arguably be included within the topoisomer category, it seems more fitting to restrict the term to full sequence inversion. Of particular interest for this work is the *re* modification (also named retro-inverso or retro-all-D by other authors, though herein we prefer *re*). As an example, Figure 1 shows the four possible arrangements a representative heptapeptide may adopt as far as sequence and chirality are concerned (the all-L but fully sequence-inverted retro version (denoted as *r* hereafter) are included for consistency). When the *re* analogue in a standard view (N-to-C-terminal from left to right) is flipped horizontally, i.e., a 180° rotation on an in-plane axis that produces a C-to-N (from left to right) arrangement, and then compared with the original peptide (all-L, N-to-C from left to right), the side chains in both structures adopt coincident orientations (Figure 1B), while the amide bond directions are reversed (NH-CO in the *re* vs. standard CO-NH in the parent) [25]. As peptide bioactivity largely involves side chain contacts, the side-chain-superimposable *re* version is a topological mimic of the parent peptide, with predictably better stability in biological media [32].

## 2. Origins of Topoisomer Peptides

Among the early attempts to explore the relationship between reversed chirality and biological activity, the work of Stewart and Wooley regarding all-D bradykinin [35,36] and of Vogler et al. regarding all-D-Val^5^-angiotensin II-Asp^1^-β-amide [35,36] is worth mentioning. In both instances, the *e* versions turned out to be completely inactive, and the outcome was rightfully interpreted in terms of a strict chiral requirement for productive receptor interaction. 

**Figure 2:**
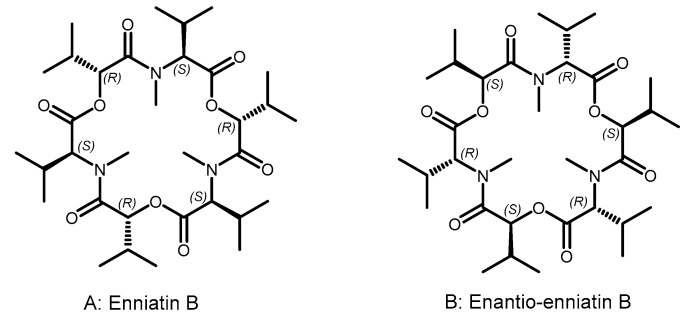
Enniatin B and its enantio form. (**A**) The configuration of enniatin B chiral centers. (**B**) Enantio-enniatin B, with the same chemical formula as enniatin B, is a mirror-image isomer with all its chiral centers reversed. Rotating enniatin B by 60 degrees in the plane illustrates the similarity.

The first successful report of a biologically active enantiomer was made in the seminal 1967 paper by Shemyakin et al. on enniatin B [37] (Figure 2), a cyclic depsipeptide (i.e., with both peptide and ester bonds) antibiotic produced by *Fusarium* fungi. The fact that synthetic enantio enniatin B was equipotent with the natural compound was rationalized as follows:

“Indeed, if one turns the formulas […] 60° in the plane of the figure, all the like asymmetric centers coincide, while each ester group will take the place of the N-methyl amide group and vice versa. […] There should therefore be a close matching of both these topochemically similar antipodes to the same stereoselective receptor…”.

By noting that a 60° rotation placed the side chains of both enantiomers pointing in the same direction (albeit with different –ester or amide– connecting units in each case), the authors were paving the way to the *re* concept of peptides topologically superimposable with the parent isomer by simple maneuvers (e.g., 60° rotation in enniatin; or 180° on an in-the-plane axis, switching N- and C-termini), and likely to be biologically active as recognizable by a chiral receptor yet plausibly longer-lived in biological media due to their D-amino acid residue contents [38].

In another significant paper published in 1969 [39], the same authors expanded their proposal to other peptide structures. Thus, [Gly^5,10^] gramicidin S, an antimicrobial peptide (AMP) active against Gram-positive bacteria, was compared with its *re* isomer and found to have comparable activity. In that same paper, Shemyakin et al. [39] recommended N- and C-terminal blocking (acetyl and carboxamide, respectively) for a linear *re* peptide to retain the activity of its parent, by attenuating electrostaticc effects of the end groups and equalizing hydrophobicity. 

In later years, similar principles of *e* and *re* switching were generalized for AMPs by the Merrifield group [38,40,41,42,43] and others [44,45], highlighting the importance of factors such as overall positive charge, amphipathicity, and serum stability for effective antimicrobial effect [46,47,48,49,50,51,52]. Again focusing on peptide–membrane interactions, in 1994, Prochiantz et al. were able to demonstrate that cell-penetrating peptides (CPPs) can enter cells independently of their chiral configuration [53]. The internalization of canonic and *e* versions of 16-residue penetratin was studied and found to be comparable, with both peptides appearing to cross the membrane through energy-independent mechanisms [53,54]. This finding has been corroborated for other CPP sequences in recent years [55,56,57,58], including CPP shuttles crossing crucial therapeutic boundaries such as the blood–brain barrier [59].

Finally, another area where topoisomeric approaches have been explored is that of peptide-based vaccines, where van Regenmortel et al. [60] demonstrated the antigenic mimicry between native peptide antigens and their *re* counterparts. In contrast to the substantial amount of work in the AMP and CPP fields, the literature does not show that these pioneering efforts have inspired a significant follow-up. 

Generalizing topoisomer strategies like those successfully described above for AMPs and CPPs to other peptide therapeutic areas such as peptide hormones has not been straightforward, underscoring the fact that, in many other cases, stereochemically stringent interactions are at play, which can be contrasted with the fairly relaxed chiral rules of engagement for membrane-active peptides. 

## 3. Membrane-Active Peptides and Their Mechanisms of Interaction

Peptide–membrane interactions arise from distinctive sequence motifs within peptides that correspond to specific physicochemical properties. Membrane-active peptides typically possess structural features (electrical charge, amphipathicity, etc.) that foster—or prevent—interaction with membrane bilayers of diverse lipid compositions. These membrane-active peptides have been categorized into two main groups based on their mode of interaction: AMPs and anticancer peptides (ACP), both functioning as membrane disruptors, and CPPs, which translocate across the membrane to enter the cell [61]. Interestingly, these groups of peptides often exhibit similar properties, with instances where AMP/ACPs overlap with CPPs, and vice versa [62]. 

**Figure 3:**
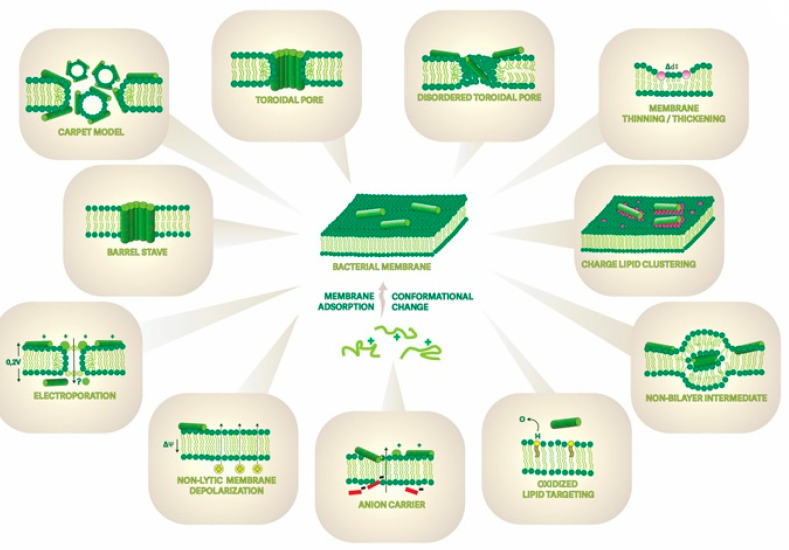
Multiple models showcase the diverse mechanisms by which AMPs disrupt bacterial membranes. In the classical view of the (not mutually exclusive) mechanisms, after an initial electrostatic interaction, peptides approach the membrane and, once they reach a critical concentration threshold, can create peptide-lined pores in the barrel-stave model, or dissolve the membrane into micellar structures in the carpet model, or establish peptide-and-lipid-lined pores in the toroidal pore model. (adapted from [68]).

As mentioned, AMPs and ACPs exert their main biological effects by disrupting or modulating the integrity and function of cell membranes [63,64]. Despite the 20,000-plus hitherto catalogued AMP/ACP sequences (from natural sources and/or the result of man-made design and production), some aspects of their mechanisms of action still stimulate research and controversy [65,66]. There is a consensus that the ability to disrupt the membrane is not only dependent on the type of AMP/ACP sequence and its conformation but also on the membrane composition of the target cells. In most typical scenarios, cationic AMP/ACPs interact with anionic phospholipid head groups on the outer leaflet of bacterial, fungal, or protozoan membranes through electrostatic and hydrophobic interactions [67]. Upon reaching a concentration threshold, the peptides fuse into the membrane and damage its structure [47,48] via processes such as those depicted in Figure 3.

**Figure 4:**
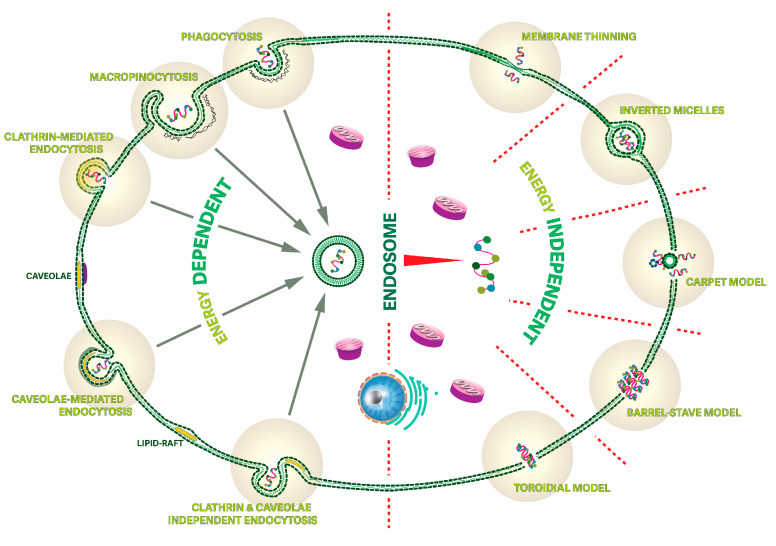
Two distinct pathways for the internalization of cell-penetrating peptides (CPPs), adapted from [87]. Endocytosis (left) involves the internalization of molecules through a process where the cell membrane engulfs them. It encompasses different subtypes, such as phagocytosis and pinocytosis, all involving expenditure of energy by the cell to internalize payloads. Non-endocytic routes (right) rely on specific interactions between the molecules involved and the cell membrane or other cellular components to facilitate uptake.

Unlike AMPs and ACPs, membrane-active CPPs enter cells via innocuously translocating membranes, without ensuing cell death [69]. This makes CPPs valuable shuttles for crossing cell membranes or other physiological barriers and intracellularly delivering diverse payloads [70,71]. The precise mechanisms of cell entry, again subject to some controversy, have nonetheless been recognized to be influenced by factors such as physico-chemical properties [72], cargo [73], concentration [74], cell type [75], temperature [76], and the environmental status of the cell [74]. There is also agreement that CPP–membrane interaction occurs primarily via either passive diffusion or endocytic or non-endocytic pathways (Figure 4). 

Passive transport is an energy-independent process that relies on the concentration gradient of a substance. It is commonly observed for ions and small molecules, including some peptides, and it is driven by the natural tendency of particles to move down concentration gradients until equilibrium is reached. This movement occurs through mechanisms such as facilitated diffusion [77,78,79,80] and osmosis [77,81,82,83]. 

Endocytosis, an energy-dependent process, involves the engulfment of molecules on a membrane to gain cell entry [84]. CPPs employ endocytosis as a frequent mechanism of internalization [84]. Endocytosis encompasses three types of processes: phagocytosis, pinocytosis, and receptor-mediated endocytosis. Pinocytosis occurs in various cell types and involves the uptake of extracellular fluid, while phagocytosis is specific to macrophages and leukocytes and entails the internalization of diverse substances. Receptor-mediated endocytosis occurs when molecular shuttles bind to specific receptors, such as in the LDL-mediated transport of cholesterol into cells. A graphical representation of these processes is shown in Figure 4 [84,85,86].

Uptake to the cytosol via energy-independent, non-endocytic pathways can be broadly categorized into two groups depending on whether or not pore formation occurs [69,74,88]. In the former case, CPPs disrupt the lipid bilayer membrane after a critical concentration is reached [89,90,91], much like the mechanism used by AMPs to disrupt bacterial membranes (see Figure 3) [48,92,93,94,95]. Additionally, certain circumstances allow CPPs and pathogenic amyloid peptides to use this mechanism for cell entry and activity (see Figure 4) [90]. Transmembrane pore mechanisms include the toroidal pore [92,96] and the barrel-stave model [48,92,96,97]. In contrast, non-pore mechanisms involve the carpet [92,96] and membrane-thinning models, among others [92,98].

## 4. Membrane-Active Topoisomeric Peptides

In designing effective membrane-active peptides, not only membrane activity but also stability in biological fluids are crucial considerations. Many promising membrane-active peptide candidates have seen their progress toward adoption on the market hampered by excessively high susceptibility to protease degradation [99,100]. As mentioned in the introduction, D-amino-acid-containing analogs of bioactive peptides, in particular those typifying *e* and *re* topoisomeric versions, may constitute viable strategies for curtailing peptide clearance via body proteases and ensuring longer systemic survival. In the following sections, we discuss in more detail examples of topoisomers within the AMP, CPP, and ACP families developed to this end.

### 4.1. AMP Topoisomers for Facing the AMR Challenge

The persistent overuse of antibiotics in both preclinical and clinical settings has contributed to an alarming rise in antimicrobial resistance (AMR). The cursory prescription of broad-spectrum antibiotics to patients with suspected infections resulted in the unwarranted use of, e.g., over 30% of prescribed antibiotics in the USA alone in 2014, with adverse effects in up to 20% of patients [101,102]. The World Health Organization (WHO) warned that, without intervention, the global consequences of AMR may be devastating, with a projected annual death toll of up to 10 million people by 2050 [103]. This prediction surpasses the combined deaths caused by cancer (8.2 million) and diabetes (1.5 million) [104].

The COVID-19 pandemic has exacerbated this issue [105,106,107,108]. According to a USA antimicrobial resistance report, 80% of patients hospitalized with COVID-19 received antibiotics [109] even though only a minority (17.6%) had a confirmed bacterial infection [110]. The financial burden on governments in relation to eventually tackling the AMRs ensuing from these practices will likely be huge. For instance, 6 out of the 18 most-worrying AMR threats have fetched up a staggering USD 4.6 bn annual bill in the USA alone [109]. Not surprisingly, deaths attributed to AMR are projected to escalate even more rapidly due to the aforementioned factors [111]. 

A major challenge in the AMR struggle revolves around the six ESKAPE bacteria (*Enterococcus faecium*, *Staphylococcus aureus*, *Klebsiella pneumoniae*, *Acinetobacter baumannii*, *Pseudomonas aeruginosa*, and *Enterobacter* spp.) [112,113,114], a group of highly virulent pathogens with an extraordinary ability to defeat antibiotic activity. In this context, AMPs are promising contenders [115,116,117] due to their rapid action at low micromolar concentrations; their broad spectrum, encompassing both Gram-positive and -negative bacteria, fungi, and viruses [92,97]; and their mechanisms of action, with a much lower tendency to develop resistance compared to conventional antibiotics [118,119]. 

AMPs were originally isolated from natural sources. Gramicidin S [120], extracted from the soil bacterium *Bacillus brevis* [121,122], was first reported in 1939. The finding of AMPs in prokaryotes raised the question of whether eukaryotes also produced AMPs against infections, particularly plants or insects lacking an immune system.

A substance in wheat flour found to be lethal to bread yeast was first described in 1896 [123], but it took some 80 years until it was isolated in a pure form (purothionin) from wheat endosperm in 1972 and shown to inhibit bacterial growth [124]. In 1962, a paper described antibacterial activity in the skin secretion of the *Bombina variegata* frog [125]; this activity was later identified as corresponding to the AMP bombinin [126]. Subsequent reports of eukaryotic AMPs included the cecropins (1981) of the *Hyalophora cecropia* moth related by Boman et al. [127,128] and, also in the 1980s, the α-defensins of rabbits [129,130,131] and humans [132] reported by Lehrer et al. and the magainins [133] from the *Xenopus laevis* frog reported by Zasloff et al. in 1987; this was followed by an ever-growing stream of AMPs from diverse sources such as β-defensins and θ-defensins from immune cells [134,135] or the first anionic (Asp rich) AMP reported in the mid-1990s [136]. At present, AMPs have been found in all types of organisms, including plants [93,137], animals [138,139,140,141,142], and bacteria [143], and they have been have widely acknowledged as ideal candidates for tackling AMR [144,145,146,147,148].

Utilizing topoisomers as natural mimics of AMPs to develop improved candidates is a sensible strategy with which to combat AMR. Among AMP topoisomers, *re* versions have a high degree of structural resemblance to natural AMPs and are therefore promising candidates. Table 1 lists the contributions to the AMP topoisomer repertoire over the last decades. 

In the 1990s, the Merrifield laboratory [42] described the *e* version of the natural AMPs cecropin A, melittin, and magainin 2 amide, as well as cecropin–melittin hybrids, and showed that the antimicrobial activity of the D-enantiomeric versions was equivalent to that of L-parental peptides. They also found that the mechanisms of action did not require a specific chiral receptor [42]. More recently, Kumar et al. studied the *e* and *re* forms of peptide 73, a derivative of aurein2.2 (GLFDIVKKVVGAL) [149,150]. Both *e*73 and *re*73 versions exhibited activity similar to that of peptide 73, including efficacy against *S. aureus* in a cutaneous infection model [150].

In a study by Lynn et al., the *e* and *re* forms of BMAP-28, a bovine cathelicidin AMP, were tested for their activity against *Leishmania* parasites. Both topoisomers effectively reduced both promastigote and amastigote forms of *L. major* [151,152]. In contrast, canonic BMAP-28 was ineffective due to degradation by the parasite metalloproteinase GP63.

Another bovine AMP, the 13-amino-acid indolicidin, isolated from neutrophil granules [153], was also studied in its *r*, *e*, and *re* versions [44]. While all the peptides exhibited antimicrobial activity comparable to that of natural indolicidin, those incorporating D-amino acids were advantageous due to their lower hemolytic activity.

Crotalicidin (Ctn), a cathelicidin AMP derived from South American pit vipers, exhibits both antibacterial and anticancer properties. Falcão et al. dissected Ctn and showed that a Ctn [15-34] fragment had similar activity but much better serum stability when compared to the parent peptide [154,155,156]. More recently [31], we investigated the *e*, *r*, and *re* topoisomers of Ctn and Ctn [15-34] and showed that while Ctn topoisomers underwent a 50% reduction in antimicrobial activity compared to the L-form, activity and improved serum stability were maintained for Ctn [15-34] topoisomers.

Neubauer et al. investigated the activity of the AMPs aurein 1.2, CAMEL, citropin 1.1, omiganan, pexiganan, and temporin A along with their *r* analogues. With the exception of *r*-omiganan, the retro analogues exhibited reduced activity compared to their native counterparts. The authors attributed the lower antimicrobial efficacy observed to a relatively higher hydrophilicity in comparison to the natural peptides. This study was useful in charting the limitations of retro analogues in antimicrobial applications, with hydrophobicity and hemolytic activity being presented as particularly relevant issues [157].

The last two examples serve to emphasize the need for caution when proposing and/or implementing topoisomeric approaches in AMPs (or, for that matter, in bioactive peptides of any type), as the biological outcomes of sequence inversion (*r* versions), D-amino acid replacement (*e* versions), or the combination of both (*re* versions) are arguably non-innocuous given their impact on D structure and ultimately activity [31,99].

**Table 1:** Examples of recent AMP topoisomers.

Name ^a^	Sequence ^b^	Topoisomer Class	Active Against	Observations ^c^	References
Retro-indolicin	RRWPWWPWKWPLI	*r*	G+ (*S. aureus*), G− (*E. coli*)	Same MIC values.	[44]
Inverso-indolicin	ilpwkwpwwpwr	*e*	G+ (*S. aureus*), G− (*E. coli*)	Same MIC values and increased stability.	[44]
Retroinverso-indolicin	rrwpwwpwkwpli	*re*	G+ (*S. aureus*), G− (*E. coli*)	Same MIC values and increased stability.	[44]
Retro-[Trp^4,6,8,9,11^Phe]-indolicidin	RRFPFFPFKFPLI	*r*	G+ (*S. aureus*), G− (*E. coli*)	Same MIC values.	[44]
Inverso-[Trp^4,6,8,9,11^Phe]-indolicidin	ilpfkfpffpfrr	*e*	G+ (*S. aureus*), G− (*E. coli*)	Same MIC values and increased stability.	[44]
Retroinverso-[Trp^4,6,8,9,11^Phe]-indolicidin	rrfpffpfkfpli	*re*	G+ (*S. aureus*), G− (*E. coli*)	Same MIC values and increased stability.	[44]
D-V_681_	kwksflktfksavktvlhtalkaiss	*e*	G+/G−	Same MIC values and increased stability.	[158]
D-V13K_D_	kwksflktfksakktvlhtalkaiss	*e*	G+/G−	Enhanced AMP activity and increased stability.	[158]
D-BMAP-28 *	GGlrslGrkilrawkkyGpiivpiiriG	*e*	*Leishmania major* (protozoa)	Enhanced AMP activity.	[151]
RI-BMAP-28 *	GiriipviipGykkwarlikrGlsrlGG	*re*	*Leishmania major* (protozoa)	Enhanced AMP activity.	[151]
D-Ano-NH_2_	Gllkriktll	*e*	G+/G−	Same MIC values.	[159]
D-GL13K	Gkiiklkaslkll	*e*	G+	Enhanced AMP activity.	[160,161]
retro-HHC10	WRIWKWWRK	*r*	G+/G−	Same MIC values.	[162]
inverso-HHC10	krwwkwirw	*e*	G+/G−	Same MIC values and increased stability.	[162]
retro-inverso-HHC10	wriwkwwrk	*re*	G+/G−	Same MIC values.	[162]
inverso-CysHHC10	ckrwwkwirw	*e*	G+/G−	Same MIC values.	[162,163,164]
IK8-all D	irikirik	*e*	G+/G−	Enhanced AMP activity.	[165]
IK12-all D	irvkirvkirvk	*e*	G+/G−	Enhanced AMP activity.	[165]
D-MPI	idwkklldaakqil	*e*	G+/G−	Same MIC values.	[166]
*r*-CAMEL	LVKLVAGIKKFLKWK	*r*	G+/G−	Curtailed AMP activity	[157]
*r*-citropin 1.1	LGGIVSAVKKIVDFLG	*r*	G+/G−	Curtailed AMP activity.	[157]
*r*-omiganan	KRRWPWWPWRLI	*r*	G+/G−	Enhanced AMP activity.	[157,167]
*r*-pexiganan	KKLIKVFAKGFKKAKKLFKGIG	*r*	G−	Same MIC values.	[157]
*r*-temporin A	LIGSLVRGILPLF	*r*	G+	Curtailed AMP activity	[157]
D-RR4	wlrrikawlrrika	*e*	G−	Same MIC values.	[168]
RI-73	lwGvwrrvidwlr	*re*	G+ (*S. aureus*)	Same MIC values.	[150]
D2D	kk(1nal)fk(1nal)knle	*e*	G+/G−	Enhanced AMP activity.	[169]
(ri)-r(P)ApoBSPro *	GsllkvprkpspiifklkGpklavhp	*re*	G−	Same MIC values and increased stability.	[170]
Ctn retro *	FPITVGIVMPKKFIKKLRKKVSKKVKKFFKKFRK	*r*	G−	Curtailed AMP activity	[31]
Ctn enantio *	krfkkffkkvkksvkkrlkkifkkpmviGvtipf	*e*	G−	Curtailed AMP activity and increased stability.	[31]
Ctn retroenantio *	fpitvGivmpkkfikklrkkvskkvkkffkkfrk	*re*	G−	Curtailed AMP activity and increased stability.	[31]
Ctn[15-34] retro *	FPITVGIVMPKKFIKKLRKK	*r*	G−	Same MIC values.	[31]
Ctn[15-34] enantio *	kkrlkkifkkpmviGvtipf	*e*	G−	Same MIC values and increased stability.	[31]
Ctn[15-34] retroenantio *	fpitvGivmpkkfikklrkk	*re*	G−	Same MIC values and increased stability.	[31]
D-Caerin	GllsvlGsvakhvlphvvpviaehl	*e*	G+	Enhanced AMP activity.	[171]

^a^ AMP name provided in the publication; ^b^ uppercase letters represent L-amino acids, while lowercase letters represent D-amino acids; ^c^ relative to original peptide; * peptides described as both AMPs and ACPs. Abbreviations: G+, Gram-positive; G−, Gram-negative; 1nal; 3-(1-naphthyl)-D-alanine; *e*, enantio; *r*, retro; *re*, retroenantio.

### 4.2. CPP Topoisomers and Drug Delivery Challenges

CPPs, also known as Trojan horse, protein translocation domains (PTD), or membrane translocation sequences (MTS), are peptides with the ability to traverse membranes —including barriers such as the gastrointestinal barrier [172], the blood–placental barrier [173], or the highly restricted blood–brain barrier (BBB) [174]—and deliver therapeutically active payloads across the boundary. CPPs are about 5–30 residues long, usually linear, (although cyclic versions have been described [27,69]), and mostly composed of L-amino acids, with some sequences including D-residues or L,D-combinations [69]. CPPs have been classified as cationic [175,176,177,178], amphipathic [179,180,181], hydrophobic [182,183,184], or anionic [185,186], with cargoes that include small molecules (drugs and dyes), proteins, nanoparticles, or genetic material [71].

Although most CPPs are considered safe, their (mostly) cationic nature may pose toxicity issues to some organs and tissues; hence it is important to develop CPPs safer for humans. Moreover, many CPPs degrade fast in biological fluids, proteolytic stability thus being critical for their efficacy as drug delivery vehicles [187,188]. The design of improved CPP platforms has addressed these issues by means of in vivo toxicity screens [189] and/or resorting to topoisomers (*e* or *re* versions) to avoid protease degradation. Some recent examples are shown in Table 2.

The first CPP identified was a fragment of the trans-activator of transcription (Tat) protein [190]. A detailed study of the protein defined Tat [48–60] as the most effective fragment [69]. Wender et al. studied a shortened version, Tat [49–57], as well as the corresponding *e* and *re* versions and found that these topoisomers were more effective than the canonic L-version in terms of entering Jurkat cells [191]. Similarly, Seisel et al. investigated the *e* and *re* versions of the Tat [48–60] sequence using iCal36, a peptide intended for patients with cystic fibrosis, as cargo, with the *re* topoisomer found to be most appropriate [192].

Another CPP discovered soon after Tat was penetratin, derived from the DNA binding domain of the Antennapedia protein [69]. Nielsen et al. showed that oral coadministration of insulin with L- or D-penetratin lowers blood glucose levels significantly more than insulin alone. D-penetratin was most effective due to its lower level of degradation by proteases [193]. Similar results in terms of blood glucose reduction were reported by Kamei et al. [194] with respect to D-PenetraMax, another *e* penetratin topoisomeric analogue.

Following the discovery of Tat and penetratin, oligoarginines such as R6 or R8 have also been recognized as effective CPPs [177]. Studies of *e/re* topoisomeric poly-Arg peptides again showed their effectiveness in penetration. For instance, Garcia et al. showed that r6 and r8 in combination with lauric acid enhanced insulin transport through the gastrointestinal tract by around 30–40% in Caco-2/HT-29 cells [195]. Similar work conducted by Kamei et al. [196] showed that r8 improved on R8 with regard to intravenous co-administration with insulin.

A rather interesting example of CPP topoisomerism is ^D^Angiopep, the *re* version of Angiopep-2, an artificial peptide that crosses the BBB [197]. By combining ^D^Angiopep with nanoparticles and a dye, effective BBB penetration and glioma targeting within the brain was demonstrated. This approach could prove particularly valuable in facilitating tumor identification during surgical procedures [198]. Another relevant BBB-crossing CPP, the THR peptide, discovered through phage display, can successfully interact with the human transferrin receptor. The protease vulnerability of the THR peptide [199] has been successfully overcome by its *re* version [28], used to transport nanoparticles inside the brain [200].

Another example worth mentioning is mastoparan (MP), a 14-residue peptide isolated from the venom of *Vespula lewisii*. Topoisomers of MP and its MitP analogue (with α-aminoisobutyric acid instead of alanine in position 10) were shown to be effective CPPs, with the *e* version of MP displaying the highest translocation efficacy and protease resistance [201].

To investigate D- and L-CPP entrance in cells, Verdurmen et al. compared the effect of three peptides (hLf, penetratin, and nonaarginine) in their canonic and *e* forms on three different cell lines (HeLa, Mc57 fibrosarcoma, and Jurkat T) [202]. They observed distinct differences in uptake efficiency between the two enantiomers at low concentrations, which could be attributed to a two-step internalization process. A first step was binding to heparan sulphates (HS) [203], the receptors of Arg-rich CPPs (especially of L-versions [202,204]), followed by internalization via endocytosis. Notably, the presence of HS appeared to hinder the efficiency of the second step for D-CPPs at lower concentrations. In contrast, at higher concentrations, D-enantiomers became more efficient, as the dominant mechanism was direct penetration [187]. These findings underscore the importance of stereochemistry, mechanisms of action, and applied concentration when studying CPPs.

Another interesting example is ^D^CDX, the *re* version of CDX, a 16-residue peptide derived from the II loop of snake neurotoxin candoxin [205], with higher transcytosis observed in BBB models compared to the protease labile L-version [206]. Han et al. proved that ^D^CDX combined with liposomes crosses the BBB in vitro, following an energy-dependent lipid raft/caveolae- and clathrin-dependent pathway [207].

Yet another example of a successful topoisomer CPP engineered into a druggable candidate, the 16-residue peptide wliymyayvaGilkrw (DRT-017), was developed by our group. It embodies the *re* version of a transmembrane (TM5) motif of the CB1 cannabinoid receptor (CB_1_R) fused with the *e* version of a BBB shuttle. The co-administration of the peptide and a cannabinoid preserves THC-induced analgesia but minimizes side effects (i.e., cognitive impairment) by restricting, both in vitro and in vivo, the formation of a heterodimer between CB_1_R and the serotonin 5HT2A receptor responsible for the unwanted side effect [208].

**Table 2:** Examples of recent CPP topoisomers.

**Name ^a^**	**Sequence ^b^**	**Cargo**	**Topoisomer Class**	**In Vitro Cell Lines Tested**	**In Vivo Models**	**References**
Tat-D	Grkkrrqrrrppq	Peptides, small molecules	*e*	Caco-2, ATCC, HTB-37, Calu-3, ATCC, HTB55	-	[192,209]
Tat*ri*	qpprrrqrrkkrG	Peptides, small molecules	*re*	Caco-2, ATCC, HTB-37, Calu-3, ATCC, HTB55	-	[192]
D-Tat 49-57	rkkrrqrrr	Small molecules, nanoparticles, proteins.	*e*	Jurkat T	-	[191,210]
D-Tat 57-49	rrrqrrkkr	Small molecules, nanoparticles, proteins.	*re*	Jurkat T, MCF-7	Peritoneal-TA3/St tumor-bearing mice	[191,210,211]
D-dfTAT	(ckrkkrrqrrrG)_2_ (disulfide bridge)	-	*e*	HeLa, MCH58 and HDF	-	[212]
D-penetratin	rqikiwfqnrrmkwkk	Insulin	*e*	Caco-2, HepG2 and IEC-6	o.a. mice	[193,194,213]
D-penetraMax	kwfkiqmqirrwknkr	Insulin	*e*	-	o.a. mice	[194]
D-polyarginine	(rrrr)_x_	Insulin, peptide, nucleic acid	*e, re*	Caco-2, HT-29, Fetal hepatocytes, HeLa, Mc57 fibrosarcoma and Jurkat T	i.v. male Sprague-Dawley rats, i.v. and i.p. ducklings	[195,196,202,214,215,216]
^D^Angiopep	yeetkfnnrkGrsGGyfft	Nanoparticles	*re*	bEnd.3 and U87	Glioma model in mice	[198,217]
THRre	pwvpswmpprht	Small molecules, nanoparticles	*re*	bEnd.3 cells	-	[28,199,200,218]
JNKD	tdqsrpvqpflnlttprkprpprrrqrrkkrG	-	*re*	Primary cortical neuronal cultures	i.p. Sprague-Dawley P7 rat pups	[216,219]
D-R9F2C	rrrrrrrrrffc	Oligonucleotides	*e*	HeLa	-	[220]
D-SAP	(vrlppp)_3_	-	*e*	HeLa	-	[221]
β-syn 36D	GvlyvGsktr	-	*e*	SH-SY5Y5	Drosophila model, mixed with the food	[222]
retro-inverso β-syn 36	rtksGvylvG	-	*re*	SH-SY5Y5	Drosophila model, mixed with the food	[222]
RI-HER-2	vcsaGftyrGepnpmseftdtnytvlapchl (cyclic disulfide form)	-	*re*	BT-474, SK-BR-3, MDA-468, and TS/A	Combination with RI-VEGF-P4, s.c. mice	[223]
RI-VEGF-P4	fsmecimrikphqGqhiGcqmti (cyclic disulfide form)	-	*re*	BT-474, SK-BR-3, MDA-468, and TS/A	Combination with RI-HER-2, s.c. mice	[223]
*i*MP	inlkalaalakkil	Peptides	*e*	U373MG	-	[201]
*r*MP	LIKKALAALAKLNI	Peptides	*r*	U373MG	-	[201]
*ri*MP	likkalaalaklni	Peptides	*re*	U373MG	-	[201]
*i*MitP	inlkklakl(Aib)kkil	Peptides	*e*	U373MG	-	[201]
*r*MitP	LIKK(Aib)ALAALAKLNI	Peptides	*r*	U373MG	-	[201]
*ri*MitP	likk(Aib)lkalkklni	Peptides	*re*	U373MG	-	[201]
*ri*DOM	qqrkrkiwsilaplGttlvklvaGic	-	*re*	Lipid vesicles	-	[224]
R.I.-p1932	qpkGppppGqpknGGqpppG	-	*re*	PE/CA PJ15 and hGF	-	[225]
NrTP5	ykqchkkGGkkGsG	-	*e*	HeLa and BHK21	-	[226]
RI-C2	arkGrsntfidc	siRNA	*re*	M17, PC12, L929, or S2103	i.v. C57BL/6 mice model	[29]
D-K4	kkkk	Peptide nucleic acid	*e, re*	Fetal hepatocytes	i.v. and i.p. ducklings	[215]
RICK	kwllrwlsrllrwlarwlG	Nanoparticles	*re*	U87	-	[227]
D-CADY-K	Glwralwrllrslwrllwk	Nanoparticles	*e*	U87	-	[227]
retro-D-HAI	hrpyiah	-	*re*	B cells	i.p. mice, s.c. rabbit	[228]
^D^VS	svafpsyrhrsfwsv	Small molecules	*re*	HUVEC and U87	i.v. intracranial tumor model mice	[229]
CHA-061	hsfriitsitlrGrrrrrrrrr	-	*re*	HK-2	Streptozotocin (STZ)-induced diabetes mouse model (via i.p. injection)	[230]
^D^CDX	GreirtGraerwsekf	Liposomes	*re*	bEnd.3 and U87	i.p. male mice	[207]
^D^PepH3	aGilkrw	Proteins, antibodies	*e*	HBEC-5i	-	[59]
DRT-017	wliymyayvaGilkrw	-	*re*	bEnd.3	i.v. mouse	[208]
^D^A7R	rpplwta	Peptides	*re*	HUVECs and U87	s.c. mice	[231]
OPBP-1	rvysf	Peptides	*re*	HUVECs and U87	s.c. mice	[231]

^a^ CPP name provided in the publication; ^b^ Uppercase letters represent L-amino acids, while lowercase letters represent D-amino acids. Abbreviations: i.v., intravenous; i.p., intraperitonial; s.c., subcutaneous; o.a., oral administration; Aib, 2-aminoisobutyric acid; *e*, enantio; *r*, retro; *re*, retroenantio.

### 4.3. ACP Topoisomers for Mitigating Side Effects in Cancer Treatments

Despite significant advances, the three main methods for cancer treatment, namely, chemotherapy [232,233,234], radiotherapy [235,236,237], and immunotherapy [235,238,239], suffer from low selectivity and serious side effects. In particular, in chemotherapy, the continued use of some antitumor drugs often gives rise to resistance [234]. Therefore, there is a clear need for new therapies that combine selective drug delivery with high toxicity against cancer cells [240].

In terms of successfully tackling the above-mentioned resistance and side effects [241], ACPs appear to be promising candidates. Currently, there are three FDA-approved ACPs, with revenues over USD 1 million [66]: goserelin (PyrHWSYs(tBu)LRP; Pyr—L-pyroglutamyl) and leuprolide (PyrHWSYlLRP), analogues of gonadotropin-releasing hormone (GnRH) [242,243]; and octreotide (fCFwLTCThre; Thre—L-threoninol), an analogue of somatostatin [244]. Since 2000, this list has expanded with entries such as ixazomib, thymalfasin, and mifamurtide [245,246].

ACPs are classified as direct- or indirect-acting based on their mechanism of action [247]. Direct-acting ACPs (DAAs) specifically target cancer cells, typically attaching to molecules that are either unique or overexpressed [248]. They are divided into five subclasses [241]: (a) CPPs acting as cytotoxic drug carriers [249], as discussed in Section 4.2.; (b) pore-forming peptides, inducing apoptosis or necrosis by interacting with phosphatidylserine anionic lipids exposed on the outer membrane of cancer cells [250]; (c) peptide inhibitors of signal transduction cascades, either inhibiting mitogenic signals or restoring the activity of tumor-suppressive proteins like p53 [251]; (d) cell-cycle-inhibitory peptides, modulating cyclin and cyclin-dependent kinase activity [252]; and (e) apoptosis-inducing peptides, inhibiting anti-apoptotic proteins from the Bcl-2 family [252].

Indirect-acting ACPs can influence the tumor environment or immune response in order to target cancer cells and are subdivided into two classes [241]: (a) immune-stimulating peptides, also referred to as peptide cancer vaccines [253], triggering immune cells such as T-cells to act as natural killers against cancer cells, and (b) analogues of hormone-releasing peptides, inhibiting the proliferation of hormone-stimulated tumor cells. These classes include the above-referred GnRH analogues goserelin and leuprolide [254,255] as well as octreotide and other somatostatin analogues [256].

Upon comparing the modes of action of pore-forming peptides, DAAs, and AMPs, it becomes clear that they exhibit similarities in terms of electrostatic interactions [257]; therefore, many AMPs tend to also be explored for their role as potential ACPs. An example, discussed in Table 1, is Ctn and its fragment Ctn [15-34]. The anti-tumor activity of both peptides towards several tumor cells has been substantiated [154], and more recently, that of their topoisomers has been, too [31]. Furthermore, the mechanism by which these peptides combat tumor cells has been elucidated. Following initial accumulation on the tumor cell surface, Ctn and Ctn [15-34] enter the tumor cell via either an endocytic pathway or an energy-independent mechanism. Ultimately, Ctn and Ctn [15-34] induce cell death through necrosis or apoptosis [258].

Table 3 presents a comprehensive collection of other topoisomeric peptides that have been explored as ACPs, regardless of their mechanisms of action.

The first, noteworthy case is that of *e* PMI. PMI is a peptide recognized for its interaction with MDM2 and MDMX, two oncoproteins that negatively regulate the functionality and stability of tumor-suppressing protein p53. Active MDM2 and MDMX cause p53 inactivation and ensuing tumor proliferation [259]. The binding of PMI to MDM2 and MDMX prevents their inhibitory action toward p53, thus ensuring that PMI can exert its tumor suppressing role. Li et al. identified three *e* peptides, ^D^PMI-α, ^D^PMI-β, and ^D^PMI-γ, capable of binding MDM2 and MDMX but unable to induce p53-dependent cell death due to their non-permeability with respect to the cell membrane [260]. They overcame this hurdle by encapsulating these peptides within liposomes decorated with an integrin-targeting cyclic-RGD peptide. This strategy allowed them to curb glioblastoma activity in vivo via the activation of the p53 pathway.

Another topoisomer ACP worth mentioning is *re* RPL (named ^D^(LPR) by the authors). RPL is the minimal structural part of CPQPRPLC, a peptide obtained via phage display-library screening [261] that binds both VEGFR-1 (vascular endothelial growth factor) and NRP-1 (neuropilin-1), two essential contributors to angiogenesis whose inhibition can lead to a decrease in tumor size. Giordano et al. developed *re* RPL as an antiangiogenic drug with promising attributes in vitro and in vivo [262]. Also, Rezazadeh et al. linked *re* RPL to technetium-99m and showed that the conjugate was a good radioligand for imaging and targeting tumors in vivo [263].

The well-known tripeptide RGD binds specifically to integrin αvβ3, making it an antiangiogenic candidate and a molecular imaging probe [264]. Ramezanizadeh et al. showed that the *re* version of RGD, i.e., dGr, in either a linear or cyclic form, was useful for tumor imaging and presented higher bioactivity than the natural version [265].

VAP is a seven-residue prostate-homing peptide that binds selectively to GRP78 (glucose-regulated protein 78) [266], which, in turn, regulates VEGF expression and is over-expressed in some tumor cells but remains absent in normal cells. Ran et al. tested *e* and *re* versions of VAP and found higher in vivo antitumoral efficacy when compared to the L-counterparts. Furthermore, tumor growth diminished with either *e*-VAP or *re-*VAP, concomitant with an increase in body weight, suggesting reduced side effects. The elevated activity of D-amino-acid-containing peptides was attributed to their resistance against proteolytic degradation [267].

As mentioned above, NRP-1 plays a pivotal role in tumorigenesis and is highly expressed within tumor cells. A library of peptides bearing the sequence motif R/K(X)_n_R/K (with the C-terminal R or K being particularly vital), named CendR, exhibited notable affinity for binding to NRP-1 [268] in the L-conformation. Upon binding, the peptides regulated vascular permeability (enhanced vascular permeability is indispensable for cancer metastasis [269]). Despite their promise, the susceptibility of CendR peptides to protease degradation resulted in low activity. However, Wang et al. [270] showed that topoisomeric CendRs retained functionality. Thus, using RGERPPR as an example, they showed that both topoisomers were superior, with the *e* version (rGerppr) displaying higher stability and stronger binding to NRP-1 than the L-peptide and the *re* version (rppreGr) demonstrating heightened tumor-penetrating prowess and stability. This outcome was interpreted using computational simulations revealing that the three D-Arg residues of the *e* version neatly aligned with the binding pockets of NRP-1, a phenomenon absent in the natural peptide [270].

A last noteworthy example of topoisomeric modulation is FP21, a 21-residue peptide (YTRDLVYGDPARPGIQGTGTF) corresponding to positions 33–53 of human follicle-stimulating hormone (FSH). The FSH receptor (FSHR) is selectively expressed in 50% to 70% of ovarian carcinomas; hence, it is a potential target in treating ovarian tumors [271,272]. Zhang et al. demonstrated that FP21, incorporated into nanoparticles, effectively bound to FSHR but suffered from a limited half-life [273]. The authors overcame this problem using the *re* version of FP21, which, again formulated as nanoparticles, achieved FSHR binding, improved biostability, and enabled a reduction in tumor size over the L-version, altogether positioning this topoisomer peptide as a promising candidate for treating ovarian cancer [274].

**Table 3:** Examples of recent ACPs topoisomers.

Name ^a^	Sequence ^b^	Topoisomer Class	In Vitro Cell Lines Tested	References
^D^PMI-α	tnwyanlekllr	*e*	U87, U251, HCT116 p53+/+ and HCT116 p53−/−	[259,275]
^D^PMI-β	tawyanfekllr	*e*	U87, U251, HCT116 p53+/+ and HCT116 p53−/−	[259,275]
^D^PMI-γ	dwwplafeallr	*e*	U87, U251, HCT116 p53+/+ and HCT116 p53−/−	[275]
^D^(LPR)	lpr	*re*	HUVEC	[262,263,276]
D-SP5	prpspkmGvsvs	*re*	SGC7901	[277]
retro-tuftsin	RPKT	*r*	A549 and HL-60	[278]
^D^(RGD)	dGr	*re*	U87MG, C6and Hela	[264,265,279]
RI-BK	rfpsfGppr	*re*	HUVEC and C6	[280]
RI-VAP	pavrtns	*re*	U87MG, HUVEC and HL7702	[267]
D-VAP	sntrvap	*e*	U87MG, HUVEC and HL7702	[267]
^D^WSW	wswGpys	*re*	U87 and HUVEC	[281]
RI-3	yr(Aib)r	*re*	RBL-2H3 and RBL-2H3/ETFR, osteosarcoma Saos-2	[282]
^D^(CendR)	rppreGr ^c^	*e*	HUVEC, C6, U87 and A549	[270]
^D^(CendR)	rGerppr ^c^	*re*	HUVEC, C6, U87 and A549	[270]
^D^T7	hrpyiah	*re*	HepG2	[283]
D-FP21	ftctGqiGprapdGyvldrty	*re*	HO8910 and HEK 293 T	[274,284]
retro-inverso FSH β 33–53 peptide	ftctkqikprapdkyvldrty	*re*	A2780	[285]
[D]-NRC-03	GrrkrkwlrriGkGvkiiGGaaldhl	*e*	HMEC, HDF and HUVEC	[286]
RIF7	rqwllfi	*re*	A549	[287]

^a^ ACP name provided in the publication; ^b^ uppercase letters represent L-amino acids, while lowercase letters represent D-amino acids; ^c^ examples of the R/K(X)_n_R/K motif discussed in the article. Abbreviations: Aib, 2-Aminoisobutyric acid; *e,* enantio; *r*, retro; *re*; retroenantio.

## 5. Conclusions

Peptides are making substantial inroads into therapeutic application in diverse fields. Peptide-based drug development has evolved from merely reproducing natural motifs to the rational engineering of peptide structures including modifications such as cyclization, stapling, conjugation, or the introduction of modified, non-coded amino acids. With over 80 peptides already on the market and many others in preclinical or clinical stages, the potential of peptides is becoming increasingly manifest across a wide range of indications [288]. A case in point is provided by combinatory anti-infective therapies, where a conventional antibiotic and an AMP are co-administered to foil antimicrobial resistance [289].

Despite such promise, therapeutic peptides continue to face significant challenges, mostly related to their stability in human fluids. In this review, we have shown that topoisomer formulations, particularly those featuring D-amino acids (*e* and *re*), often provide viable alternatives to canonical L-versions. This must not be misconstrued, however, as implying that topoisomers do not face challenges in their scope and applications. For instance, retro peptides can present flexibility and adaptability issues that require cautious structural scrutiny [157]. Additionally, enantio versions, particularly when used as CPPs, may also present limitations intrinsically linked to factors such as stereochemistry, mechanisms of action, or applied concentration [202]. These and other limitations notwithstanding, e.g., the higher price (at least twice) of D- compared to L-amino acids, the cost-effectiveness of topoisomer-based approaches is likely to pay off over time and thus furnish valuable tools for advanced peptide-based therapies.

## Data Availability

The data can be shared up on request.
